# Supplementary material for: Non-targeted N-glycome profiling reveals multiple layers of organ-specific diversity in mice
Source: Nat Commun. 2024 Nov 9;15:9725. doi: 10.1038/s41467-024-54134-z (PMC11550822; doi:10.1038/s41467-024-54134-z)
Supplement: Supplementary file 1 — Supplementary Information [file 41467_2024_54134_MOESM1_ESM.pdf]

Supplementary Information

Non-targeted N-glycome profiling reveals multiple layers of organ-specific diversity in mice.

Johannes Helm[1*], Stefan Mereiter[2,3*], Tiago Oliveira[2,3], Anna Gattinger[3,4], David M. Markovitz[5], Josef M. Penninger[2,3,6,7], Friedrich Altmann[1], Johannes Stadlmann[1,8].

Affiliations

1. Institute of Biochemistry, Department of Chemistry, University of Natural Resources and Life Sciences (BOKU), Muthgasse 18, 1190 Vienna, Austria.
2. Eric Kandel Institute, Department of Laboratory Medicine, Medical University of Vienna, Spitalgasse 23, 1090 Vienna, Austria.
3. Institute of Molecular Biotechnology of the Austrian Academy of Sciences (IMBA), Vienna BioCenter (VBC), Dr. Bohr-Gasse 3, 1030 Vienna, Austria.
4. Bioinformatics Research Group, University of Applied Sciences Upper Austria, Softwarepark11, 4232 Hagenberg, Austria.
5. Division of Infectious Diseases, Department of Internal Medicine, and the Programs in Immunology, Cellular and Molecular Biology, and Cancer Biology, University of Michigan, Ann Arbor, MI 48109, USA.
6. Department of Medical Genetics, Life Sciences Institute, University of British Columbia, Vancouver Campus, 2350 Health Sciences Mall, Vancouver, BC Canada V6T 1Z3.
7. Helmholtz Centre for Infection Research, Braunschweig, Germany
8. BOKU Core Facility Mass Spectrometry, University of Natural Resources and Life Sciences (BOKU), Muthgasse 18, 1190 Vienna, Austria.

*These authors contributed equally

Table of contents:

Supplementary Figures

Supplementary Figure 1. Precursor-independent MS/MS-based N-glycome profiling of 20 mouse tissues

Supplementary Figure 2. Comparative N-glycome profiling of 20 mouse tissues

Supplementary Figure 3. Impact of SNOG-score filtering on data quality

Supplementary Figure 4. Impact of SNOG-score filtering on the total ion current (TIC) and number of unique mass bins across all samples

Supplementary Figure 5. Impact of SNOG-score filtering on data quality of the liver 1 data set

Supplementary Figure 6. Empiric determination of the cut-off values for the extended SNOG-scores (eSNOG)

Supplementary Figure 7. Tissue specific expression of specific N-glycan modifications

Supplementary Figure 8. MS/MS supported identification of a N-glycan with the composition $Hex_6HexNAc_6Fuc_4GlcA_1$ carrying a fucosylated HNK-1 epitope in kidney

Supplementary Figure 9. Profiling the isomeric complexity of the murine N-glycome

Supplementary Figure 10. Additional isomeric profiling of the murine N-glycans.

Supplementary Tables

Supplementary Table 1. MS/MS-based N-glycome profiling – numeric spectral counting results.

Supplementary Table 2. Overview over diagnostic fragment ions used for sub-structural profiling of the mouse N-glycome

Supplementary Table 3. Overview mouse tissue samples used in this study

Supplementary Notes

Supplementary Note 1. Calculation of the SNOG-score

Supplementary Note 2. Calculation of the extended SNOG-scores (eSNOG-score)

Supplementary Figures

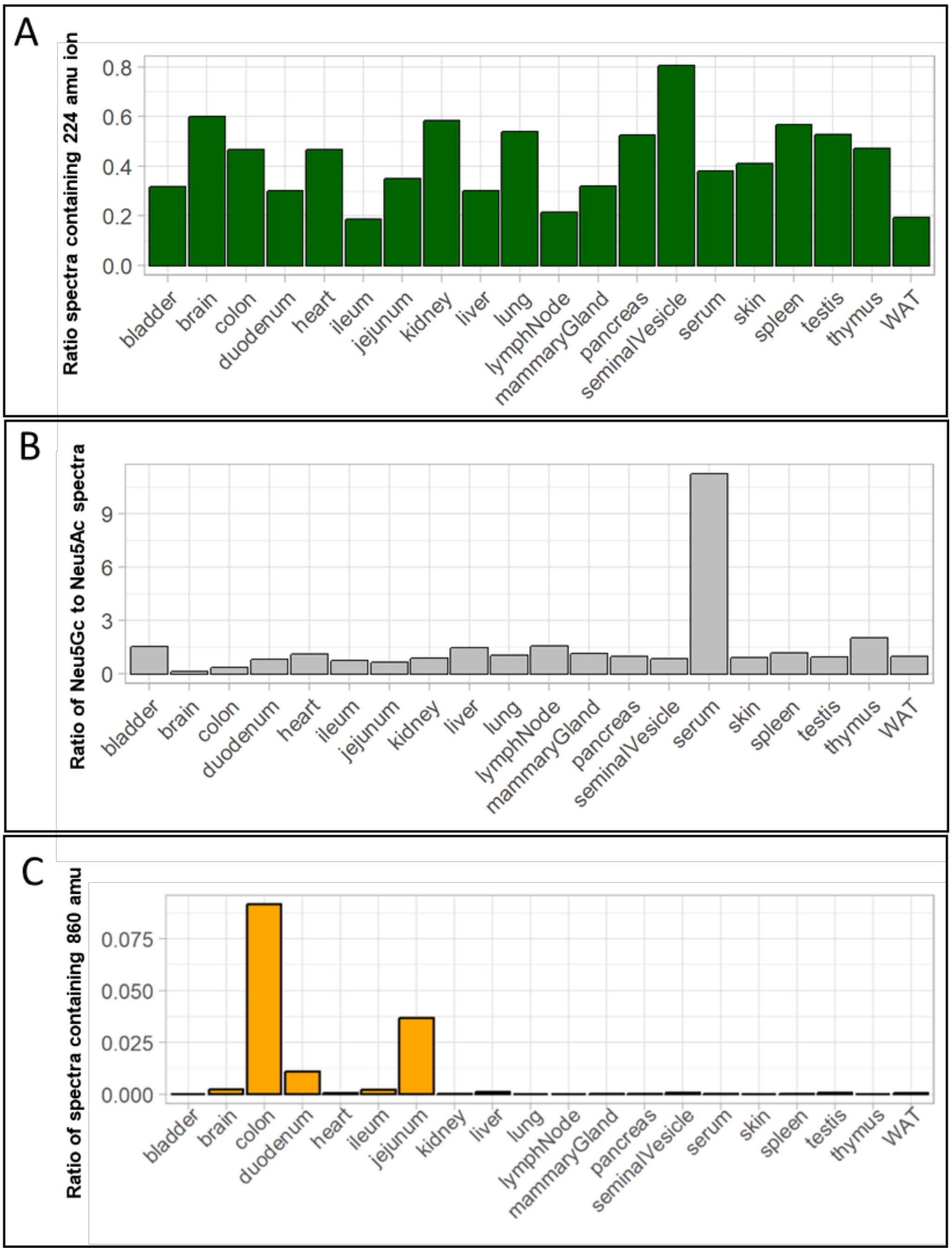

Supplementary Figure 1. Precursor-independent MS/MS-based N-glycome profiling of 20 mouse tissues. (A) Relative abundance of MS/MS spectra containing the glycan-specific fragment ion 224.1 amu (reduced HexNAc). **(B)** Ratio of spectra containing the diagnostic fragment ion for Neu5Gc (290.1 amu) and spectra containing the diagnostic fragment ion for Neu5Ac (274.1 amu). **(C)** Ratio of spectra containing the fragment ion indicative for the Sda-antigen (860.3 amu). Only N-glycan associated spectra are considered (hence containing the fragment ion 224.1 amu). WAT - white adipose tissue.

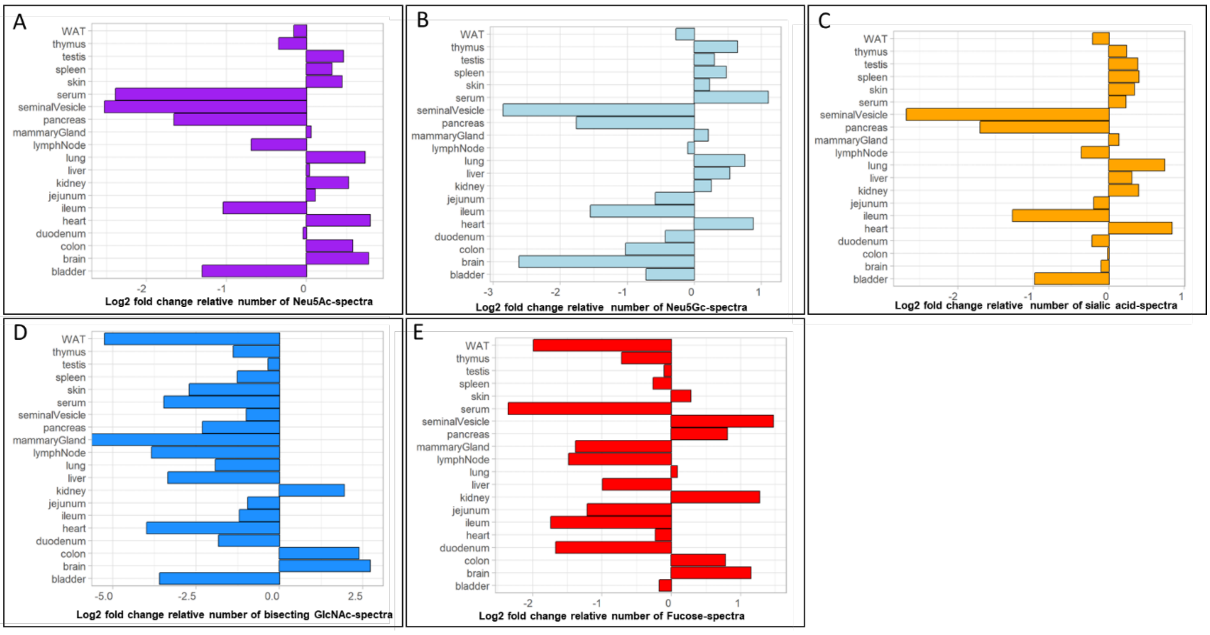

Supplementary Figure 2. **Comparative N-glycome profiling of 20 mouse tissues**. The graphs show the log2-fold change of the number of MS/MS spectra containing a specific diagnostic fragment ions in relation to the mean across all tissues. **(A)** Log2-fold change of the number of MS/MS containing a Neu5Ac-specific fragment ion (274.1 amu) **(B)** Log2-fold change of the number of MS/MS containing a Neu5Gc-specific fragment ion (290.1 amu). **(C)** Log2-fold change of the number of MS/MS containing a Neu5Ac- or Neu5Gc-specific fragment ion (274.1 amu or 290.1 amu)**. (D)** Log2-fold change of the number of MS/MS containing a bisecting GlcNAc-specific fragment ion (792.3 amu). **(E)** Log2-fold change of the number of MS/MS containing a fucose-specific fragment ion (512.2 amu). Because of gas-phase re-arrangement of fucose-residues, this fragment ion does not discriminate between core- and distal fucose. Only N-glycan associated spectra (i.e. containing the fragment ion 224.1 amu) were considered. WAT - white adipose tissue.

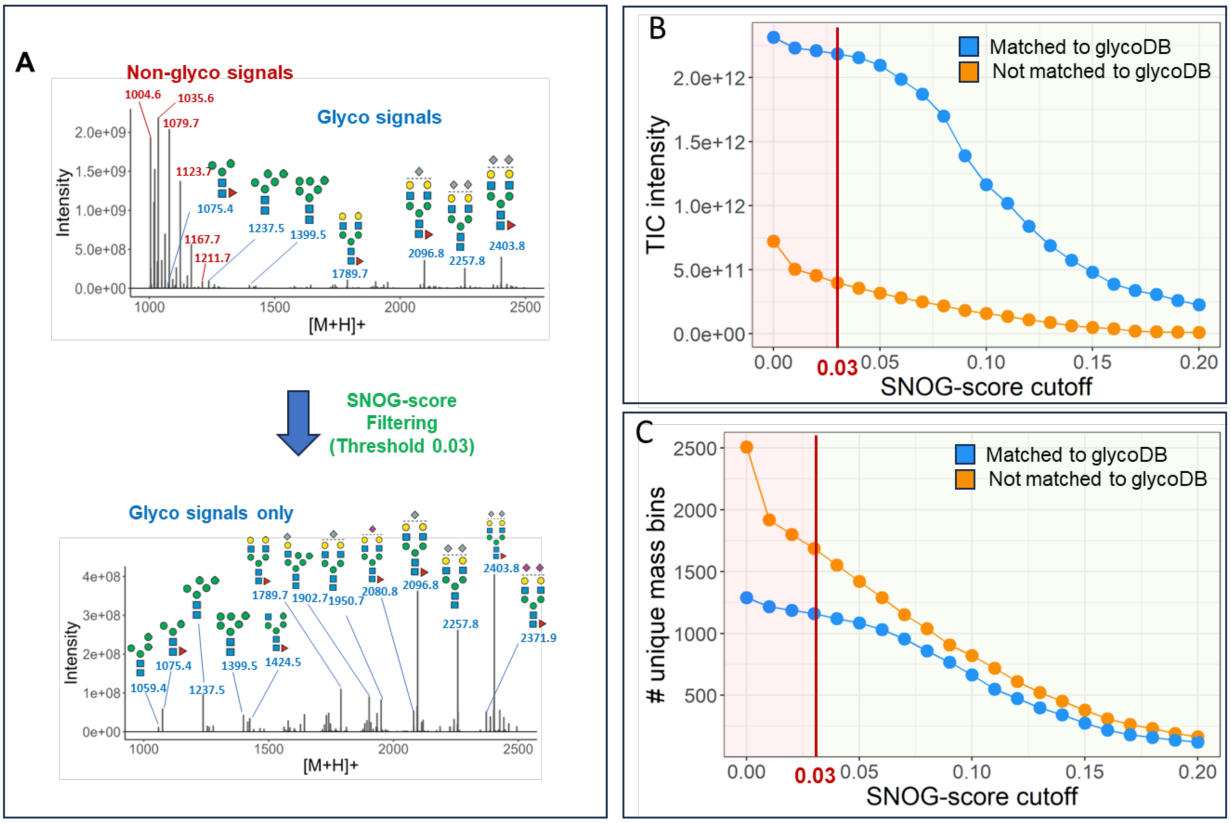

Supplementary Figure 3. Impact of SNOG-score filtering on data quality. (A) SNOG-filtering of the lymph node 1 data set. Filtering effectively removes signals derived from contaminants and retains N-glycan associated signals. **(B)** Impact of different SNOG-score cutoff values on the accumulated total ion current (TIC) of all analyzed samples. **(C)** Impact of different SNOG-score cutoff values on the number of unique mass-bins across all analyzed samples. Blue dots represent values corresponding to mass bins which have an entry in our glycoDB (including sodium and ammonium adducts), orange dots represent values which have no entry in our glycoDB. N-glycan cartoons depict tentative structure assignments based on composition, using the Symbol Nomenclature for Glycans (SNFG). WAT - white adipose tissue.

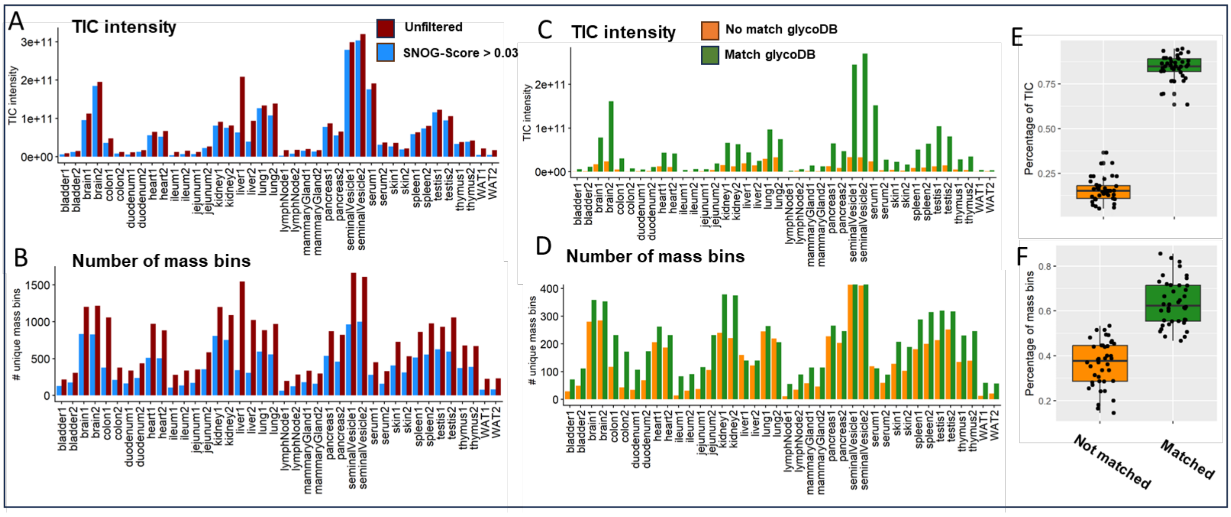

Supplementary Figure 4. Impact of SNOG-score filtering on the total ion current (TIC) and number of unique mass bins. (A) Impact of the SNOG-score filtering with a cutoff at 0.03 on the TIC, stratified across all samples. Red bars represent the TIC of the unfiltered data, blue bars represent the filtered data. **(B)** Impact of the SNOG-score filtering with a cutoff at 0.03 on the number of unique mass bins, stratified across all samples. Red bars represent the TIC of the unfiltered data, blue bars represent the filtered data. **(C)** Distribution of the TIC of the SNOG-score filtered data set (> 0.03) categorized into mass bins that have an entry in our glycoDB (green) and into mass bins which have no entry in our glycoDB (including sodium and ammonium adducts (orange) across all samples**. (D)** Distribution of the number of unique mass bins of the SNOG-score filtered data set (> 0.03) categorized into mass bins that have an entry in our glycoDB (green) and into mass bins which have no entry in our glycoDB (including sodium and ammonium adducts) (orange) across all samples. **(E)** Boxplots of the percentage compositions of the TIC derived from mass bins which have an entry in our glycoDB (including sodium and ammonium adducts) (green) or have no entry in our glycoDB (orange) across all samples. **(F)** Boxplots of the percentage compositions of the number of unique mass-bins which have an entry in our glycoDB (including sodium and ammonium adducts) (green) or have no entry in our glycoDB (orange) across all samples. WAT - white adipose tissue.

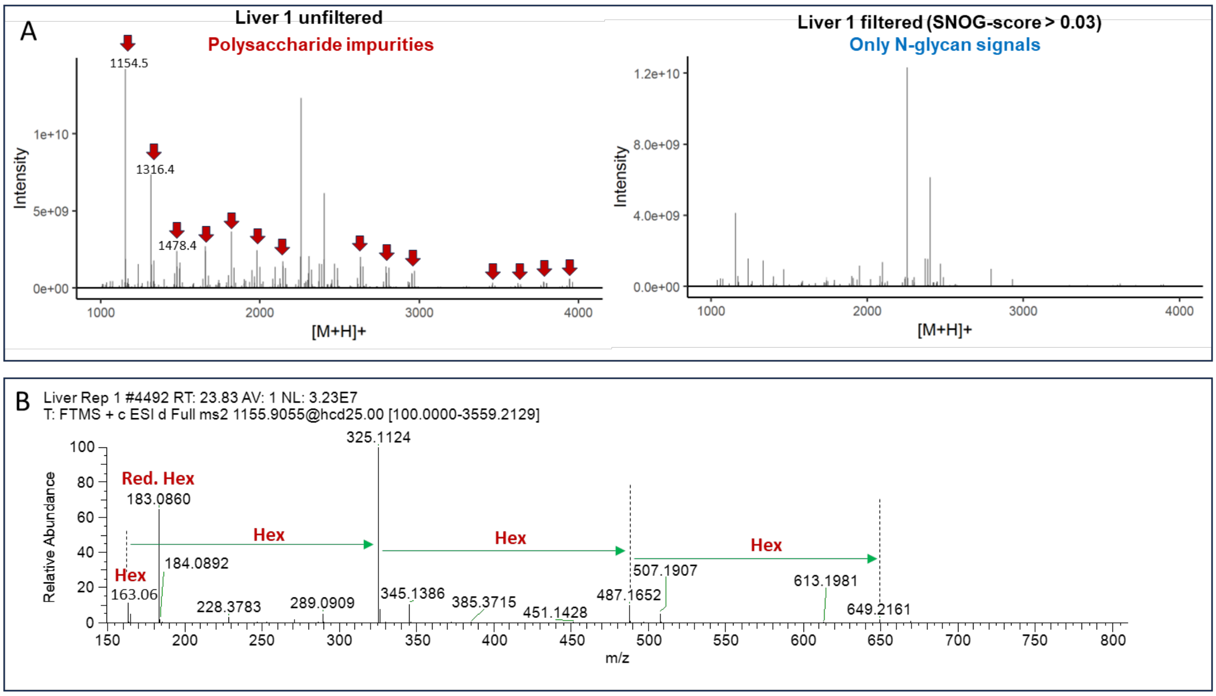

Supplementary Figure 5. **Impact of SNOG-score filtering on data quality of the liver 1 data set. (A)** Comparison between the unfiltered (left) and filtered liver 1 data set (right). The red arrows depict signals derived from polysaccharide impurities commonly observed in N-glycan preparations. The SNOG-score filter efficiently removes impurity-derived signals. (**B**) Exemplary MS/MS spectrum of a polysaccharide impurity derived from the liver 1 data set. The absence of a 224.1 amu diagnostic fragment ion leads to the removal of the respective precursor mass.

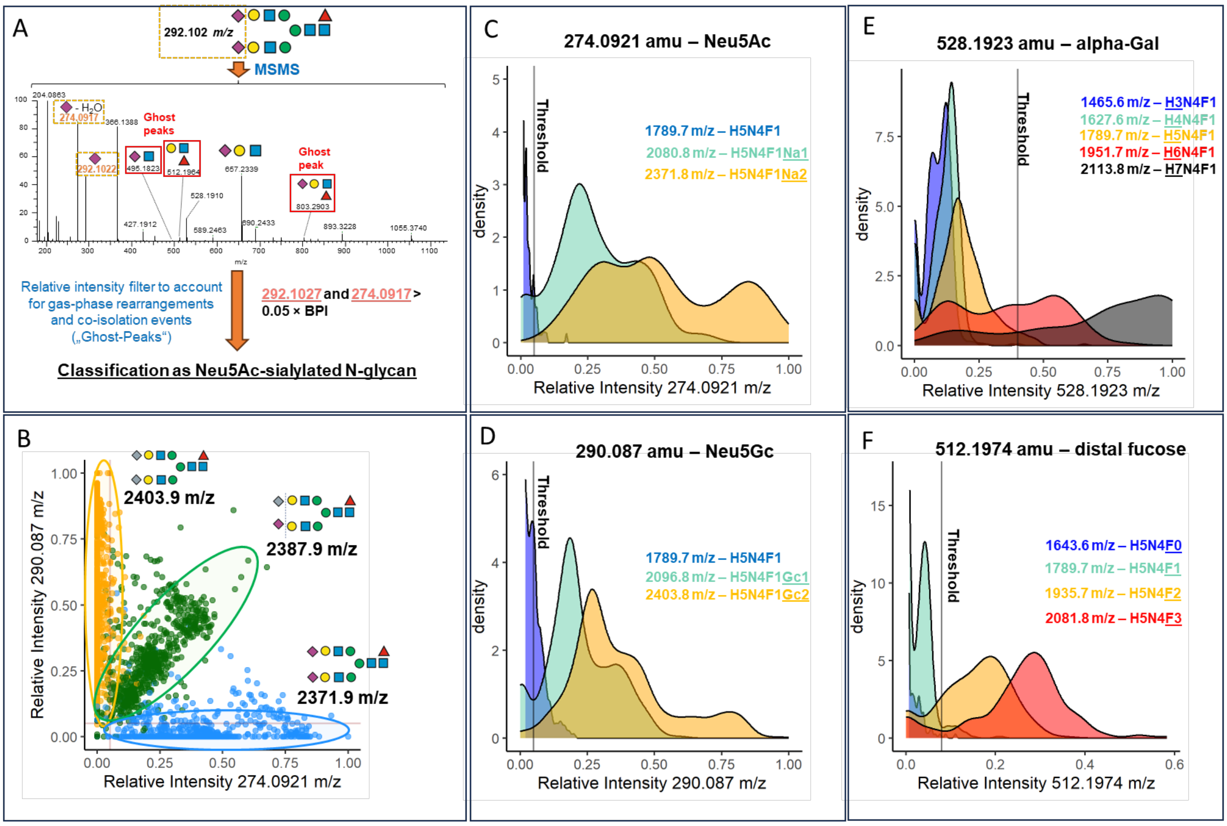

Supplementary Figure 6. Empiric determination of the cut off values for the extended SNOG-scores (eSNOG). (A) Representative MS/MS spectrum of a Neu5Ac-sialylated N-glycan with the composition $Hex_{5}HexNAc_{4}Fuc_{1}Neu5Ac_{2}$. Red boxed fragment ions are the result of gas-phase rearrangements of Neu5Ac or fucose residues. The simultaneous presence of both the 292.1 amu and 274.1 amu fragment ions are used to classify precursors as "Neu5Ac-sialylated". **(B)** Relative Intensities of the Neu5Gc-specific fragment ion (290.1 amu), and the Neu5Ac-specific fragment ion (274.1 amu) derived from three different N-glycan precursors, i.e., $Hex_{5}HexNAc_{4}Fuc_{1}Neu5Gc_{2}$ (orange dots), $Hex_{5}HexNAc_{4}Fuc_{1}Neu5Ac_{1}Neu5Gc_{1}$ (green dots), and $Hex_{5}HexNAc_{4}Fuc_{1}Neu5Ac_{2}$ (blue dots) across all samples. **(C)** Density plot of the relative intensity of the 274 .1 amu fragment ion diagnostic for Neu5Ac-sialylated N-glycans for three different N-glycan compositions, i.e., $Hex_{5}HexNAc_{4}Fuc_{1}$ (blue), $Hex_{5}HexNAc_{4}Fuc_{1}Neu5Ac_{1}$ (turquoise), and $Hex_{5}HexNAc_{4}Fuc_{1}Neu5Ac_{2}$ (orange) across all tissues. The plot is cut-off at a density value of 5. An $eSNOG_{274}$-score of 0.05 efficiently discriminated Neu5Ac-sialylated N-glycans. **(D)** Density plot of the relative intensity of the 290.1 amu fragment ion, diagnostic for Neu5Gc-sialylated N-glycans, for three different N-glycan compositions, i.e., $Hex_{5}HexNAc_{4}Fuc_{1}$ (blue), $Hex_{5}HexNAc_{4}Fuc_{1}Neu5Gc_{1}$ (turquoise), and $Hex_{5}HexNAc_{4}Fuc_{1}Neu5Gc_{2}$ (orange) across all tissues. The plot is cut-off at a density value of 6. An $eSNOG_{290}$-score of 0.05 efficiently discriminated Neu5Gc-sialylated N-glycans. **(E)** Density plot of the relative intensity of the 528.2 amu fragment ion for five different compositions (i.e., $Hex_{3}HexNAc_{4}Fuc_{1}$ (blue), $Hex_{4}HexNAc_{4}Fuc_{1}$ (turquoise), $Hex_{5}HexNAc_{4}Fuc_{1}$ (orange), $Hex_{6}HexNAc_{4}Fuc_{1}$ (red), $Hex_{7}NAc_{4}Fuc_{1}$ (black), and $Hex_{7}HexNAc_{4}Fuc_{1}$ across all tissues. With some exceptions, $Hex_{6}HexNAc_{4}Fuc_{1}$ (red) and $Hex_{7}NAc_{4}Fuc_{1}$ (black) are considered to contain alpha galactose. An $eSNOG_{528}$-score of 0.35 efficiently discriminated alpha-galactosylated N-glycans. **(F)** Density plot of the relative intensity of the 512.2 amu fragment ion, diagnostic for antenna fucosylation, for four different compositions (i.e., $Hex_{5}HexNAc_{4}$ (blue), $Hex_{5}HexNAc_{4}Fuc_{1}$ (turquoise), $Hex_{5}HexNAc_{4}Fuc_{2}$ (orange), and $Hex_{5}HexNAc_{4}Fuc_{3}$ (red) across all tissues. With some exceptions, the composition $Hex_{5}HexNAc_{4}Fuc_{1}$ (turquoise) is considered core fucosylated. An $eSNOG_{512}$-score of 0.075 efficiently discriminated antenna-fucosylated N-glycans and accounts for the potential gas-phase rearrangement of core fucose residues.

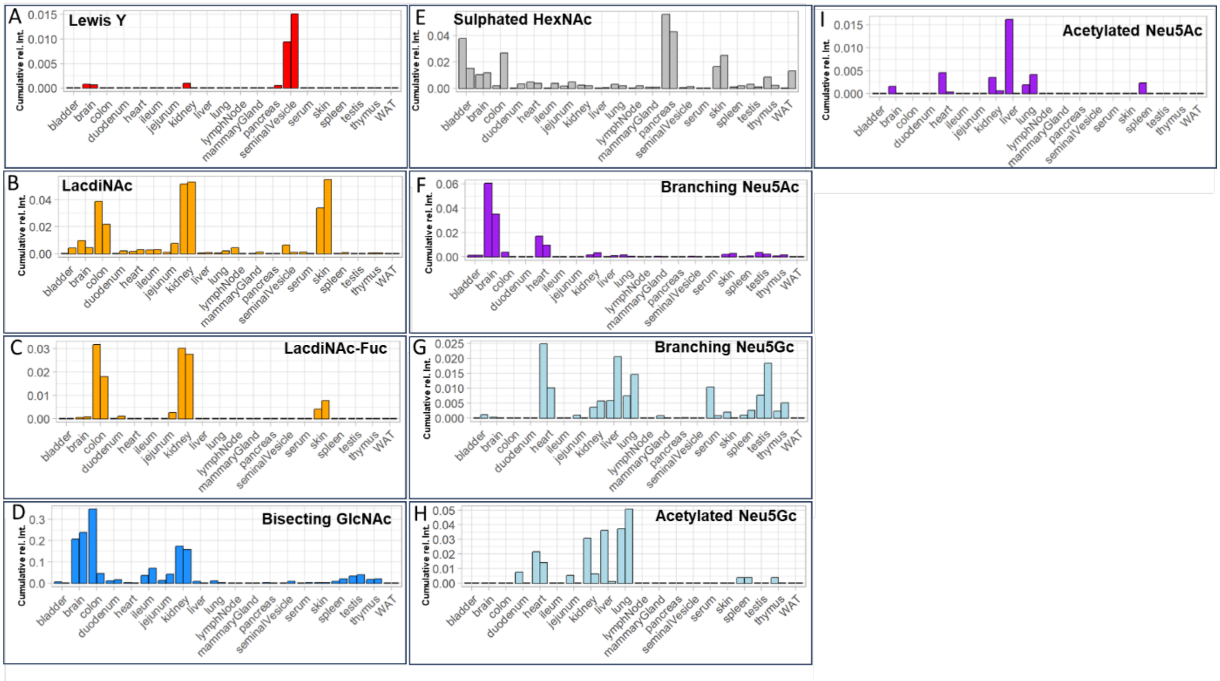

Supplementary Figure 7. Tissue specific expression of specific N-glycan modifications. (A) Lewis Y epitope. Comparative analysis of N-glycans carrying the Lewis Y epitope across tissues. **(B)** LacdiNAc modification. Comparative analysis of N-glycans carrying the LacdiNAc modification across tissues. (**C**) Fucosylated LacdiNAc modification. Comparative analysis of N-glycans carrying the fucosylated LacdiNAc modification across tissues. (**D**) Bisecting GlcNAc. Comparative analysis of N-glycans carrying a bisecting GlcNAc across tissues. **(E)** Sulfated HexNAc. Comparative analysis of N-glycans carrying sulfated GlcNAc across tissues. (**F**) Branching Neu5Ac. Comparative analysis of N-glycans carrying branching Neu5Ac across tissues. **(G)** Branching Neu5Gc. Comparative analysis of N-glycans carrying branching Neu5Gc across tissues. **(H)** Acetylated Neu5Gc. Comparative analysis of N-glycans carrying acetylated Neu5Gc across tissues. **(I)** Acetylated Neu5Ac. Comparative analysis of N-glycans carrying acetylated Neu5Ac across tissues. All values are normalized to the total glyco-TIC of the respective tissue. WAT - white adipose tissue.

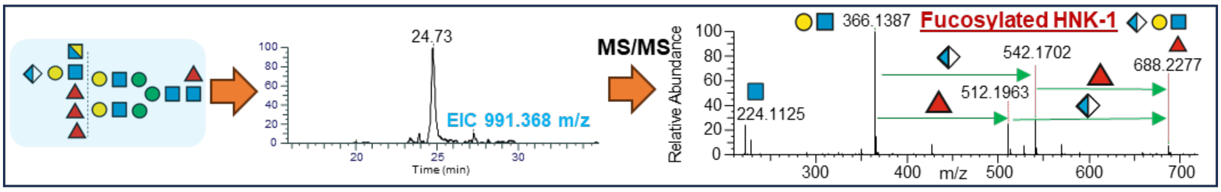

Supplementary Figure 8. MS/MS supported identification of a N-glycan with the composition $Hex_6HexNAc_6Fuc_4GlcA_1$ carrying a fucosylated HNK-1 epitope in kidney.

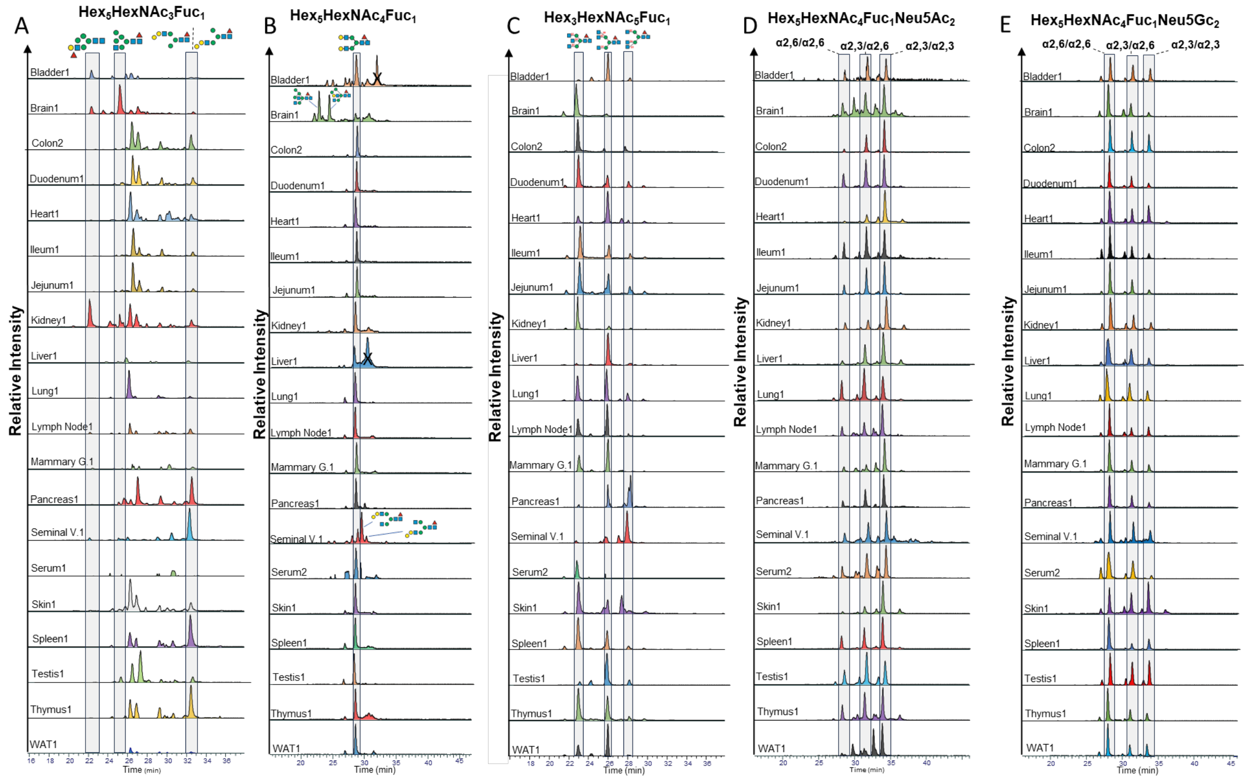

Supplementary Figure 9. Profiling the isomeric complexity of the murine N-glycome. The highly isomer-selective stationary phase porous graphitic carbon (PGC) was used to separate even closely related isomers. Already existing N-glycan retention libraries combined with MS/MS data were used to identify the exact structures of the respective isomers. All retention times were normalized to the retention time of the ubiquitous Man5 N-glycan. **(A)** shows the elution profile for the composition $Hex_5HexNAc_3Fuc_1$ across all analyzed tissues. **(B)** shows the elution profile for the composition $Hex_5HexNAc_4Fuc_1$ across all analyzed tissues. **(C)** shows the elution profile for the composition $Hex_3HexNAc_5Fuc_1$ across all analyzed tissues. **(D)** shows the elution profile for the composition $Hex_5HexNAc_4Fuc_1Neu5Ac_2$ across all analyzed tissues. **(E)** shows the elution profile for the composition $Hex_5HexNAc_4Fuc_1Neu5Gc_2$ across all analyzed tissues.

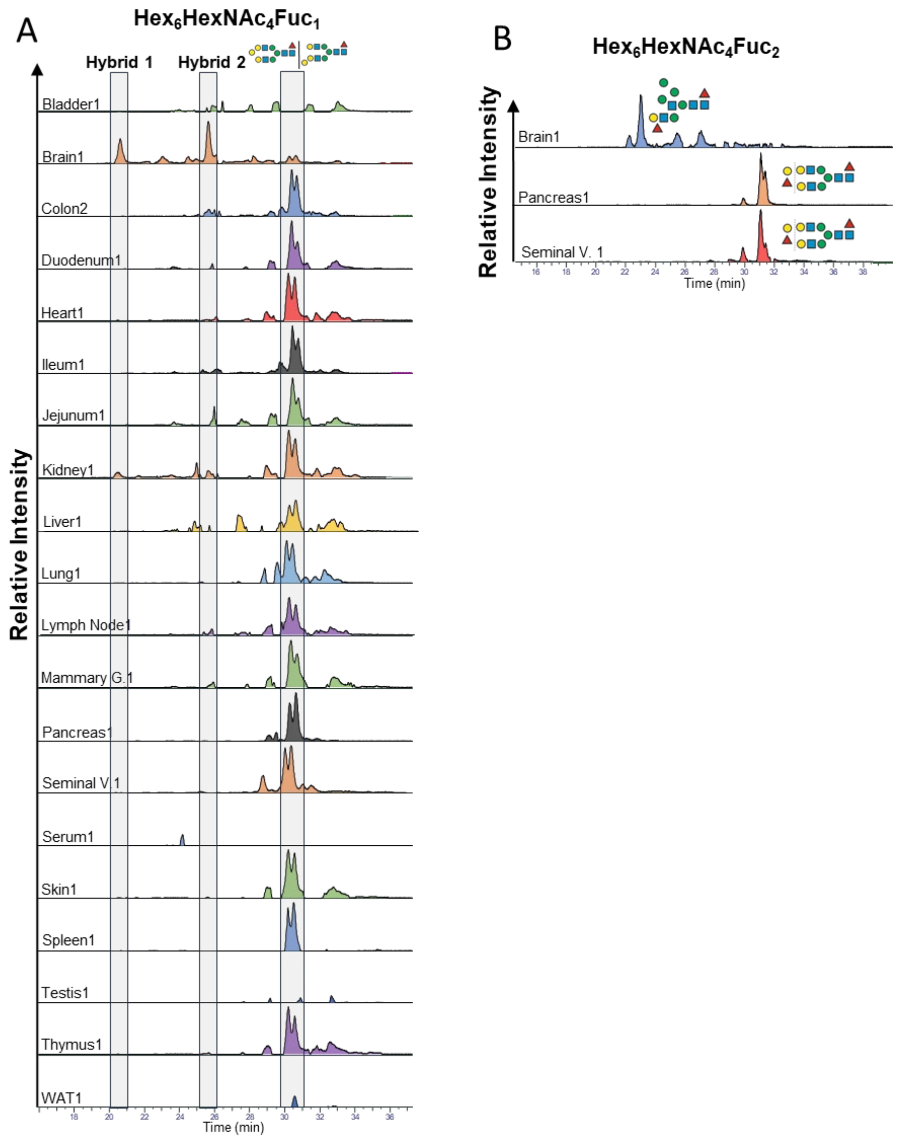

Supplementary Figure 10. Additional isomeric profiling of the murine N-glycans. The highly isomer-selective stationary phase porous graphitic carbon (PGC) was used to separate even closely related isomers. Already existing N-glycan retention libraries combined with MS/MS data were used to identify the exact structures of the respective isomers. All retention times were normalized to the retention time of the ubiquitous Man5 N-glycan. **(A)** shows the elution profile for the composition $Hex_6HexNAc_3Fuc_1$ across all analyzed tissues. **(B)** shows the elution profile for the composition $Hex_6HexNAc_4Fuc_2$ across brain, pancreas, and seminal vesicle. WAT - white adipose tissue.

Supplementary Tables

Supplementary Table 1. MS/MS-based N-glycome profiling – Numeric spectral counting results.

Tissue	Replicate	Total number of MS/MS spectra	Glyco-associated	Neu5Ac-associated	Neu5Gc-associated	Fucose-associated	SDA-associated	Bisecting GlcNAc-associated
WAT	1	9151	1853	507	1853	129	0	0
	2	9371	1678	460	1678	138	0	0
Bladder	1	8952	2513	317	2513	409	0	0
	2	8941	3114	585	3114	592	0	0
Brain	1	13691	7556	5180	7556	5740	11	227
	2	13943	8983	6159	8983	6322	27	504
Colon	1	11920	6341	4781	6341	4117	963	234
	2	12768	4874	2006	4874	1806	57	10
Duodenum	1	12490	2870	819	2870	199	22	1
	2	12866	4728	1757	4728	492	43	9
Heart	1	12753	6228	3874	6228	1474	3	2
	2	12502	5518	3193	5518	1333	0	1
Ileum	1	12305	2013	293	2013	151	5	5
	2	12048	2475	453	2475	201	3	5
Jejunum	1	12444	3121	1056	3121	236	108	2
	2	13547	5886	2633	5886	924	191	29
Kidney	1	14147	8032	4118	8032	6350	1	233
	2	13568	8069	4266	8069	6220	1	191
Liver	1	14913	4741	1271	4741	552	3	0
	2	13686	3808	1472	3808	591	5	0
Lung	1	13503	8002	4980	8002	2211	0	16
	2	13472	6494	4019	6494	2221	0	10
Lymph Node	1	8566	1271	190	1271	116	0	0
	2	10336	2719	632	2719	271	0	0
Mammary Gland	1	10094	3312	1094	3312	365	1	0
	2	10488	3221	1271	3221	414	0	0
Pancreas	1	13959	7520	911	7520	3380	0	11
	2	13493	6815	1150	6815	3773	0	1
Seminal Vesicles	1	14848	11716	767	11716	10576	7	13
	2	14855	12181	1149	12181	10951	11	34
Serum	1	12924	5348	462	5348	340	0	3
	2	8826	2866	139	2866	127	0	1
Skin	1	12932	5231	2441	5231	1780	0	6
	2	12454	5127	2385	5127	1444	0	2
Spleen	1	13283	7280	3832	7280	1845	0	17
	2	13551	7910	4373	7910	2263	1	25
Testis	1	14581	7475	4309	7475	2247	7	54
	2	14554	7840	4167	7840	2186	3	24
Thymus	1	12653	5720	1805	5720	1167	0	15
	2	13303	6437	2076	6437	1081	0	17

Supplementary Table 2. Diagnostic fragment ions and associated eSNOG-score cutoffs used for sub-structural profiling of N-glycans.

Modification	Diagnostic fragment ions [H^+] [amu]	eSNOG-score cutoff
Neu5Ac	292.1072 & 274.0921	0.025 & 0.05
Neu5Gc	308.0976 & 290.087	0.025 & 0.05
branching Neu5Ac	495.1821	0.025
branching Neu5Gc	511.177	0.025
Neu5Ac-associated di-sialyl Lewis C	495.1821 & 948.3303	0.025 & 0.005
Neu5Gc-associated di-sialyl Lewis C	511.177 & 980.3201	0.025 & 0.005
Acetylated Neu5Ac	334.1133 & 316.1027	0.005 & 0.005
Acetylated Neu5Gc	350.1082 & 332.0976	0.005 & 0.005
Antennary fucose	512.1974	0.075
Alpha-galactose	528.1923	0.35
HNK-1 epitope	542.1716	0.05
Bisecting GlcNAc	792.3234	0.025
LacdiNAc	407.1661	0.04
Fucosylated LacdiNAc	407.1661 & 553.224	0.04 & 0.01
Lewis Y	512.1974 & 658.2553	0.2 & 0.07
Sulphated HexNAc	284.0435	0.01
Reduced N-glycan	224.1118	0.03

Supplementary Table 3. Overview mouse tissue samples used in this study.

Organ	Female	Male	Extracted section or part	Opened and cleaned with PBS
Ear skin	n = 2		Slice	
Pancreas	n = 2		Whole	
Duodenum	n = 2		Middle section	Yes
Jejunum	n = 2		Middle section	Yes
Ileum	n = 2		Middle section	Yes
Colon	n = 2		Middle section	Yes
Spleen	n = 2		Whole	
Kidney	n = 2		Left	
Liver	n = 2		Whole	
Brain	n = 2		Whole	
White adipose tissue	n = 2		Epididymal	
Mammary glands	n = 2		Inguinal mammary fat pad excluding lymph node	
Lymph node	n = 2		Inguinal	
Thymus	n = 2		Whole	
Testicle		n = 2	Left	
Bladder		n = 2	Whole	Yes
Serum		n = 2	Submandibular bleeding	
Seminal vesicle		n = 2	Left	

Supplementary Notes

Supplementary Note 1. Calculation of the SNOG-score

The SNOG-score is a metric utilized to effectively differentiate signals originating from N-glycans from those originating from contaminants, such as polysaccharides. In experiments involving porous graphitic carbon (PGC)-LC-MS/MS, N-glycans are commonly reduced. The resulting reduced GlcNAc fragment ion (224.1 amu) serves as a diagnostic marker for N- (or O)-glycans. To compute the SNOG-score specific to mass bins (0.01 Da mass bins), the mean relative intensity of the 224.1 amu fragment ion is calculated across all MS/MS spectra within each precursor mass bin. This calculation is performed on a per-sample basis across all detected mass bins. Mass bins with a SNOG-score exceeding 0.03 are selected to construct a sample-specific target list, from which precursor-intensity information is retrieved.

Supplementary Note 2. Calculation of the extended SNOG-scores

The extended SNOG-scores (eSNOG) serve to categorize N-glycans based on sub-structural characteristics such as Neu5Ac-sialylation, antenna-fucosylation, or GlcNAc-sulfation. These scores are calculated in a similar manner to the original SNOG-score. Specifically, the mean relative intensity of one or more diagnostic fragments, which are specific to the modification of interest, is calculated across all MS/MS spectra within the corresponding precursor mass bin. This calculation is performed on a per-sample basis across all identified mass bins. The cutoff values for different eSNOG-scores vary depending on the specific modification and have been determined empirically (Supplementary Table 2).
