# Supplementary material for: Non-targeted N-glycome profiling reveals multiple layers of organ-specific diversity in mice
Source: Nat Commun. 2024 Nov 9;15:9725. doi: 10.1038/s41467-024-54134-z (PMC11550822; doi:10.1038/s41467-024-54134-z)
Supplement: Supplementary file 3 — Description of Additional Supplementary Files [file 41467_2024_54134_MOESM3_ESM.pdf]

Description of Additional Supplementary Files

Non-targeted N-glycome profiling reveals multiple layers of organ-specific diversity in mice.

Johannes Helm[1*], Stefan Mereiter[2,3*], Tiago Oliveira[2,3], Anna Gattinger[3,4], David M. Markovitz[5], Josef M. Penninger[2,3,6,7], Friedrich Altmann[1], Johannes Stadlmann[1,8].

Affiliations

1. Institute of Biochemistry, Department of Chemistry, University of Natural Resources and Life Sciences (BOKU), Muthgasse 18, 1190 Vienna, Austria.
2. Eric Kandel Institute, Department of Laboratory Medicine, Medical University of Vienna, Spitalgasse 23, 1090 Vienna, Austria.
3. Institute of Molecular Biotechnology of the Austrian Academy of Sciences (IMBA), Vienna BioCenter (VBC), Dr. Bohr-Gasse 3, 1030 Vienna, Austria.
4. Bioinformatics Research Group, University of Applied Sciences Upper Austria, Softwarepark11, 4232 Hagenberg, Austria.
5. Division of Infectious Diseases, Department of Internal Medicine, and the Programs in Immunology, Cellular and Molecular Biology, and Cancer Biology, University of Michigan, Ann Arbor, MI 48109, USA.
6. Department of Medical Genetics, Life Sciences Institute, University of British Columbia, Vancouver Campus, 2350 Health Sciences Mall, Vancouver, BC Canada V6T 1Z3.
7. Helmholtz Centre for Infection Research, Braunschweig, Germany
8. BOKU Core Facility Mass Spectrometry, University of Natural Resources and Life Sciences (BOKU), Muthgasse 18, 1190 Vienna, Austria.

*These authors contributed equally

Supplementary Data Files

Supplementary Data 1. “SampleParameterFile.xml”

Description: DeCon2 parameter file used in this study.

Supplementary Data 2. “Annotated_MassList.csv”

Description: Annotated Mass List of theoretical N-glycan precursor mass-bins and the respective intensity values, sample specifically.

Supplementary Data 3. “full N-glycome.jpg”

Description: Semi-quantitative histograms of SNOG-filtered LC-MS data of all samples.

Supplementary Software Files

Supplementary Software File 1. “HelmJ_MereiterS_Supplementary Software_MS2Oxoplot.zip”

Description: MS2_oxoplot.pl is a software tool implemented in the cross-platform programming language Perl. The script sequentially reads in all .mgf-files in a user-defined directory/folder, extracts the intensity values of user-defined m/z values (+/- a user-defined mass-tolerance) and puts them out to .mgf-file specific .csv files (automated file name generation: INPUT_FILENAME_RepIntensities.csv). Next to the actual script, the .zip folder contains a README file, a sample input-file for testing (mouseNglycome_brain_2.mgf) and an expected sample output-file (mouseNglycome_brain_2_RepIntensities.csv)
