# Supplementary figures and images for: Non-targeted N-glycome profiling reveals multiple layers of organ-specific diversity in mice
Source: Nat Commun. 2024 Nov 9;15:9725. doi: 10.1038/s41467-024-54134-z (PMC11550822; doi:10.1038/s41467-024-54134-z)

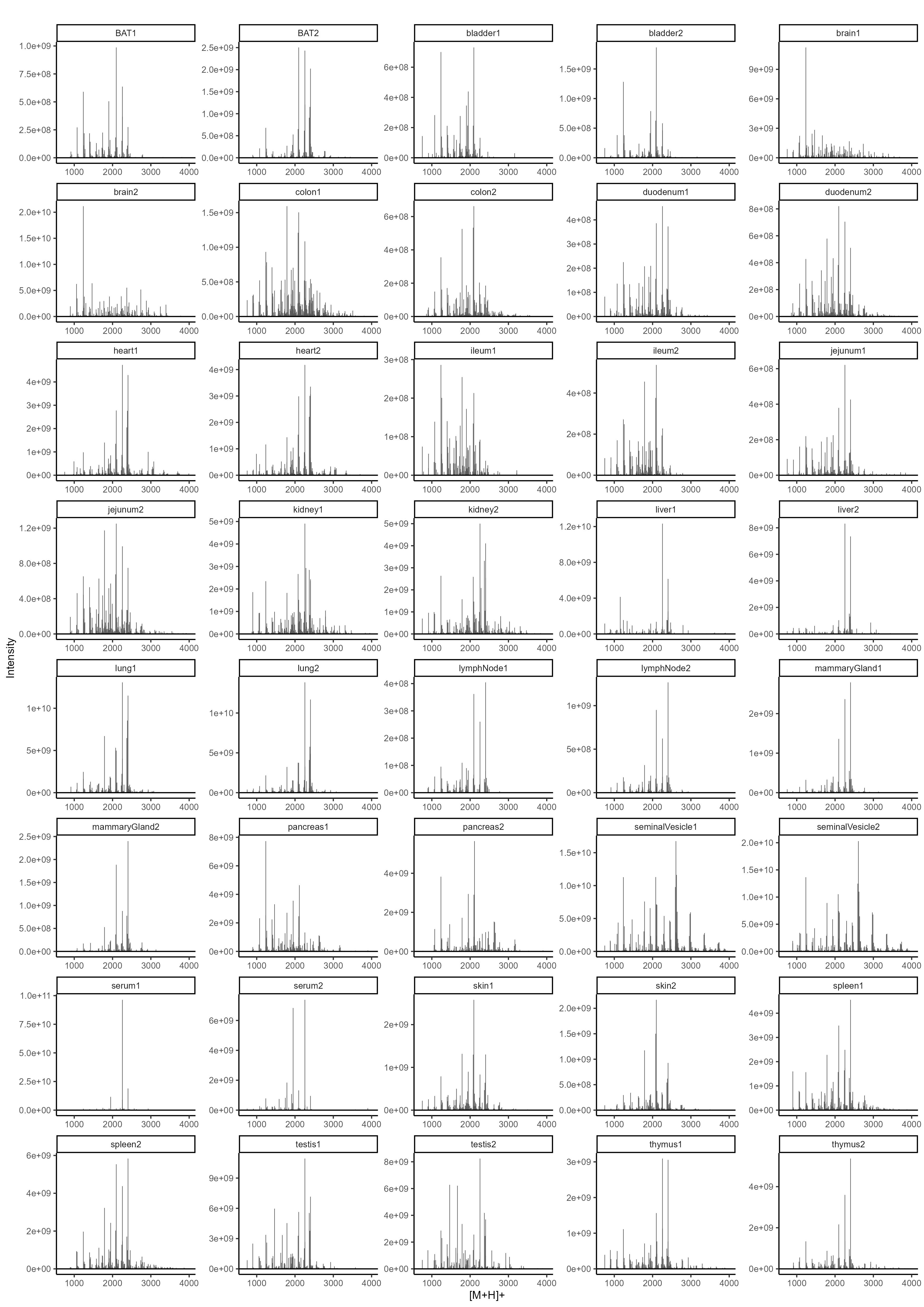

Supplement: Supplementary file 6 — Supplementary Data 3 [file 41467_2024_54134_MOESM6_ESM.jpg]
